# Beninese children with cerebral malaria do not develop humoral immunity against the IT4-VAR19-DC8 PfEMP1 variant linked to EPCR and brain endothelial binding

**DOI:** 10.1186/s12936-015-1008-5

**Published:** 2015-12-08

**Authors:** Sofia Nunes-Silva, Sébastien Dechavanne, Azizath Moussiliou, Natalia Pstrąg, Jean-Philippe Semblat, Stéphane Gangnard, Nicaise Tuikue-Ndam, Philippe Deloron, Arnaud Chêne, Benoît Gamain

**Affiliations:** Inserm UMR_1134, Paris, France; Université Paris Diderot, Sorbonne Paris Cité, UMR_S1134, Paris, France; Institut National de la Transfusion Sanguine, 6 rue Alexandre Cabanel, 75015 Paris, France; Laboratory of Excellence GR-Ex, Paris, France; Institut de Recherche pour le développement, UMR_216, Mère et enfant face aux infections tropicales, Paris, France; Faculté de pharmacie, PRES Sorbonne Paris Cité, Paris, France

**Keywords:** Endothelial protein C receptor, *var* genes, *Plasmodium falciparum* erythrocyte membrane protein 1, Cerebral malaria, *Plasmodium falciparum*, Immunity

## Abstract

**Background:**

Malaria is still one of the most prevalent infectious diseases in the world. Sequestration of infected erythrocytes (IEs) is the prime mediator of disease. Cytoadhesion of IEs is mediated by members of the highly diverse *Plasmodium falciparum* erythrocyte membrane protein 1 (PfEMP1). A restricted sub-set of *var* genes encoding for PfEMP1s possessing the domain cassettes DC8 and DC13 were found to bind to the endothelial protein C receptor (EPCR). These *var* genes were shown to be highly expressed by parasites from patients with severe malaria clinical outcomes compared to those from patients with uncomplicated symptoms.

**Methods:**

In order to further study the molecular mechanisms underlying DC8/DC13 expressing IEs adhesion to EPCR, a method was developed to produce highly pure recombinant EPCR. The IT4 parasite strain was selected on either anti-IT4-VAR19 purified IgG, EPCR or human brain endothelial cell line and their *var* gene expression profiles as well as their binding phenotypes were compared. The N-terminal region of IT4-VAR19 comprising a full-length DC8 cassette as well as the single EPCR binding CIDRα1.1 domain were also produced, and their immune recognition (IgG) was assessed using plasma samples from Beninese children presenting acute mild malaria, severe malaria or cerebral malaria at the time of their admission to the clinic, and from convalescent-phase plasma collected 30 days after anti-malarial treatment.

**Results:**

The multi-domain VAR19-NTS-DBLγ6 binds to EPCR with a greater affinity than the CIDRα1.1 domain alone and this study also demonstrates that VAR19-NTS-DBLγ6 binding to the EPCR-expressing endothelial cell line (HBEC5i) is more pronounced than that of the CIDRα1.1 domain alone. IT4-VAR19 represents the preferentially expressed-PfEMP1 when FCR3-IEs are selected based on their capability to bind EPCR. Notably, no significant difference in the levels of antibodies towards IT4-VAR19 antigens was observed within all clinical groups between plasma samples collected during the acute malaria phase compared to samples collected 30 days after anti-malaria treatment.

**Conclusions:**

These data indicate that even being the preferentially selected IT4-EPCR-binding variant, the IT4-VAR19-DC8 region does not appear to be associated with the acquisition of antibodies during a single severe paediatric malaria episode in Benin.

**Electronic supplementary material:**

The online version of this article (doi:10.1186/s12936-015-1008-5) contains supplementary material, which is available to authorized users.

## Background

Malaria is still one of the most prevalent infectious diseases in the world, affecting 198 million individuals per year and causing an estimated 584,000 subsequent deaths, mostly in children aged under 5 years accounting for 78 % of all deaths [1]. *Plasmodium falciparum* is responsible for the most severe malaria cases and fatal conditions. *P. falciparum* has developed an efficient immune evasion strategy in which antigenic variation associated with cytoadhesion mechanisms play a central role.

Indeed, the ability of *P. falciparum* infected erythrocytes (IEs) to adhere to host receptors, such as CD36, chondroitin sulfate A (CSA) and ICAM-1 present on the surface of microvascular endothelial cells or on the syncytiotrophoblast lining the intervillous placental blood space [2], allows IE sequestration and prevents IE transit through the spleen’s red pulp and their subsequent retention and clearance [3, 4]. Sequestration is the prime mediator of disease, creating blood flow obstructions and damage to the endothelial barrier, inducing a cascade of inflammatory and coagulation pathways [2].

Cytoadhesion of IEs is mediated by members of the highly diverse *P. falciparum* erythrocyte membrane protein 1 (PfEMP1) encoded by approximately 60 *var* genes per parasite genome [5, 6]. A single *var* gene is expressed at a time and the corresponding PfEMP1 is exported at the IE’s surface. Switching between variants allows the exposure of different antigenic determinants to the immune system and rapid changes in IE receptor tropism [6, 7].

Although the *var* repertoires are highly divergent, genes can be classified into three main groups (A, B and C) and two intermediate groups B/A and B/C based on their upstream promoter sequence (Ups), their chromosomal location/transcriptional direction and their coding region organization [8, 9]. All PfEMP1 variants display a N-terminal segment (NTS) followed by successive Duffy-binding-like (DBL) and cysteine-rich interdomain region (CIDR) domains [10]. Analysis of almost 400 PfEMP1 sequences revealed conserved domain structures permitting a sub-division of these putative functional groups into 18 well-defined domain cassettes (apart from *var3*, *var1csa* and *var2csa* which are relatively conserved between different parasite genomes) [11].

Recently, a small sub-set of chimeric *var* genes belonging to the group B/A (group B Ups and group A coding sequences) has been linked to cerebral malaria. Indeed, IEs expressing these genes were preferentially selected after consecutive panning rounds either on the human brain endothelial cell line HBEC-5i or on primary culture of human brain microvascular endothelial cells [12, 13]. Furthermore, a restricted sub-set of *var* genes encoding PfEMP1s possessing the domain cassettes (DC) DC8 and DC13 were found to be expressed at a higher level in patients with severe malaria clinical outcomes compared to patients presenting uncomplicated symptoms [14].

The DC8 cassette is composed of four domains (DBLα2-CIDRα1.1-DBLβ12-DBLγ4/6) whereas DC13 contains only two domains (DBLα1.7-CIDRα1.4). Both DC8 and DC13 expressing IEs have recently been shown to bind to a specific receptor on the endothelium, the endothelial protein C receptor (EPCR), via their CIDRα1.1 or CIDRα1.4 domain [15]. These non-CD36 binding PfEMP1 variants were shown to avidly bind to diverse brain endothelial cells but also to endothelial cells originating from other tissues [16]. Notably, the four individual domains from DC8 are able to bind to endothelial cells [16]. Since DC8-CIDRα1.1 is, to date, the only EPCR-binding domain of the cassette, these results suggest that DC8-PfEMP1 binding to endothelial cells rely on other types of interaction, implicating one or several, yet unidentified, receptor(s).

The identification of EPCR as an IEs receptor opened new avenues that might help understanding the pathogenesis of severe malaria. Several studies, sometimes conflicting, exploring the link between EPCR and malaria pathophysiology, revealed the complexity of how *P. falciparum* may interact with its host.

Autopsies performed on Malawian children who died from cerebral malaria exposed a loss of EPCR in cerebral microvessels at the site of IE sequestration [17]. This might at first appear contradictory with the potential role of EPCR as a major anchor point for IEs in the brain, but the precise sequence of events leading to the post-mortem observations remains unknown. EPCR promotes cytoprotection and anti-inflammatory signaling in a variety of cell types via its interactions with activated protein C (APC) [18]. The local loss of EPCR may, therefore, implicate coagulation and inflammation at the IE sequestration site, leading to a detrimental outcome. Furthermore, DC8-PfEMP1 has been shown to compete the binding of APC to EPCR [15], most likely exacerbating the inflammatory cascades.

A recent study showed that Thai malaria patients carrying a mutation in the PROCR gene that leads to higher levels of soluble EPCR in the plasma, were significantly protected against severe malaria compared to other PROCR genotypes [19]. Nevertheless, some contrasting data suggest that genetic diversity in the PROCR gene is not linked with severe malaria outcome in Ghanaian children [20] and that high plasma levels of soluble EPCR are associated with increased mortality in children in Benin [21].

In this context, it is evident that additional work is needed to understand the clinical consequences of IE binding to EPCR and to decipher the detailed cooperative molecular mechanisms (including sequestration, inflammation, coagulation) implicated in childhood cerebral malaria and their relative importance in disease severity.

The PfEMP1 N-terminal region appears to have an important role in IE cytoadhesion, but it is not clear if all DC8 cassettes present the same binding properties, nor if different EPCR-binding IEs have equivalent severe disease potential. Sequestration may be mediated by a multi-adhesive phenomenon involving numerous endothelial adhesion receptors and parasite ligands in order to maximize adhesion.

In this study aiming at assessing the importance of DC8/DC13 expressing IEs in EPCR binding, erythrocytes infected with the IT parasite strain were selected on either anti-PfEMP1 IT4-VAR19 purified IgG, EPCR or on the human brain endothelial cell line HBEC-5i and their *var* gene expression profiles as well as their binding phenotypes were compared. Furthermore, the N-terminal region of PfEMP1 IT4-VAR19 comprising a full-length DC8 cassette as well as the single EPCR binding domain CIDRα1.1, produced as recombinant proteins, were used to analyse their recognitions by plasma IgG from Beninese children presenting acute mild malaria, severe malaria or cerebral malaria at the time of their admission to the clinic and 30 days later. Taken together, the data indicate that IT4-VAR19 is the preferentially selected IT4-EPCR-binding *var* gene, but that humoral immunity against the EPCR binding VAR19-DC8 cassette or the CIDRα1.1 domain is not boosted during a single paediatric severe malaria episode in Benin.

## Methods

### Expression and purification of recombinant proteins

A synthetic gene sequence encoding the N-terminal region of IT4-VAR19 (VAR19-NTS-DBLγ6, residues G2-P1713) [Uniprot: A3R6S3] was designed with optimized codon usage for heterologous expression in human-based cell lines. This gene was cloned into a pTT3 vector with an N-terminal murine Ig κ-chain leader sequence and a hexa-His C-terminal tag. VAR19-NTS-DBLγ6 was produced as a soluble and secreted recombinant protein. Expression was carried out in the permanent cell line established from primary embryonic human kidney, HEK293F (Life Technologies), as already described [22]. The protein was purified on a HisTrap High Performance Ni-affinity column (GE Healthcare), followed by gel filtration chromatography on a Superdex 200 10/300 GL column (GE Healthcare).

The gene sequence encoding VAR19-CIDRα1.1 (residues S580-P739, boundaries based on [23]) was amplified by PCR from IT4 genomic DNA and cloned into a modified pET28a vector in which a sequence coding an N-terminal HA-tag (YPYDVPDYA) has been inserted after the NcoI restriction site. The recombinant VAR19-CIDRα1.1 protein was expressed in the SHuffle^®^ strain (NEB Biolabs) of *Escherichia coli* as a soluble protein after IPTG induction at 20 °C for 20 h. Cells were then centrifuged, resuspended in 20 mM Tris–HCl, 150 mM NaCl, pH 7.5 and lysed by three successive passages in an Emulsiflex homogenizer (Avestin). VAR19-CIDRα1.1 was purified on a HisTrap High Performance Ni-affinity column (GE Healthcare), followed by a gel filtration chromatography on a Superdex 200 10/300 GL column (GE Healthcare).

The gene encoding soluble EPCR (residues S18-S210) [Uniprot: Q9UNN8] was amplified by PCR from a human lung endothelium cDNA library. The EPCR gene sequence was cloned into two different pTT3 vectors, a pTT3 vector with an N-terminal murine Ig κ-chain leader sequence and a hexa-His C-terminal tag and a modified pTT3 vector possessing a FLAG tag just upstream of the hexa-His C-terminal tag. Both EPCR recombinant proteins were produced in HEK293F cells [22] and purified on a HisTrap High Performance Ni-affinity column (GE Healthcare), followed by a gel filtration chromatography on a HiLoad 16/60 Superdex 75 column (GE Healthcare).

A qualitative analysis of all recombinant proteins was performed by SDS-PAGE (Coomassie blue staining) and Western blotting with relevant antibodies.

### Human brain endothelial cell culture

The human brain endothelial cell line HBEC5i (CDC, Atlanta, USA) was cultured in DMEM/F-12 GlutaMAX medium (Gibco) supplemented with 10 % heat-inactivated fetal bovine serum (Gibco), 1X endothelial cell growth factor (Sigma) and 10U/ml penicillin/streptomycin (Gibco).

### Parasite culture and infected erythrocyte selection

The *P. falciparum* laboratory adapted parasite line FCR3 (IT4) was grown in O^+^ human erythrocytes in RPMI 1640 medium containing Hepes (25 mM) and l-glutamine (2 mM) (Gibco) supplemented with 5 % Albumax, 5 % human serum, 0.1 mM hypoxanthine and 20 µg/ml gentamicin [24]. Parasites were routinely genotyped by PCR using MSP1/MSP2 primers [25] and tested for potential mycoplasma contamination (LookOut Mycoplasma PCR Detection Kit by SIGMA). Cultures were routinely selected by gelatin flotation using Plasmion (Fresenius Kabi) to maintain knob-positive parasites [26]. Synchronized parasite cultures (3–6 % parasitaemia) at mid/late trophozoite stages were purified using VarioMACS and CS columns (Miltenyi Biotec) as previously described [27].

Erythrocytes infected with FCR3 (IT4) were selected for the CSA-binding phenotype by multiple panning rounds on CSA. These CSA-binding IEs are referred to as FCR3-CSA throughout this paper.

IEs FCR3-VAR19 and FCR3-EPCR were selected on purified rabbit anti-IT4-VAR19 antibodies and EPCR, respectively. FCR3-CSA was used as the starting culture for carrying out the different selection processes. One-hundred µl of Dynabeads (‘Protein G’ for rabbit antibody selection or ‘His-tag Isolation and Pulldown’ for EPCR selection) were coated with 20 µg of purified anti-VAR19 antibodies or 20 µg of EPCR for 10 min, washed twice with PBS and blocked with PBS 1 % BSA for 10 min at room temperature (RT). Purified IEs at trophozoite stage were then allowed to adhere to the coated beads for 20 min at 37 °C. The unbound IEs were washed out with PBS while the bound IEs were isolated using magnetic force and brought back into culture dishes. The day after panning, the beads were removed from the cultures and RNA was extracted for *var* transcription profile analysis. Six to seven rounds of panning were needed to obtain a population in which the *var* gene transcription profile did not vary any more, reflecting complete selection for a given binding phenotype.

### Animal immunization

Immunization with VAR19-NTS-DBLγ6 recombinant protein was performed by BIOTEM, France, according to animal immunization guidelines. In brief, two New Zealand White rabbits received 50 µg of recombinant protein in FREUND adjuvant intradermally for the first immunization followed by three subcutaneous boosts (at day 14, 28 and 42) of 25 µg of protein. Sera were collected before immunization (Pre-Immune) and at day 49 and 63 according to the immunization schedule. IgG were purified from rabbit sera using HiTrap Protein G High Performance columns (GE Healthcare). Purified antibodies were dialyzed against PBS 1X pH 7.2. Antibody titres for each sample were determined by ELISA using the immunizing proteins as target antigens. The titres were calculated using 4-parameter curve fitting and represent the serum dilution at which 50 % of the maximum recognition signal was reached.

### ELISA-based binding inhibition assay

ELISA plates (Nunc) were coated with 100 µl of recombinant VAR19-NTS-DBLγ6 protein (10 µg/ml in PBS) and incubated overnight at 4 °C. After coating, the wells were blocked with PBS 1 % BSA 0.05 % Tween (PBST-BSA), 150 µl per well at 37 °C for 1 h. After removing the blocking solution, serial dilutions of purified anti-VAR19 antibodies (concentrations ranging from 0.18 to 1.8 µg/ml diluted in PBST-BSA) were added to the wells and the plates were incubated at 37 °C for 1 h. Wells were then washed three times with PBST-BSA and 1 µg/ml of EPCR was added for 1 h at 37 °C. The wells were washed with PBST and EPCR-binding was detected with a HRP-conjugated mouse anti-FLAG M2 antibody (Abcam), diluted 1:2000 in PBST-BSA. Plates were read at 655 nm after addition of 100 µl of TMB (3,3′,5,5′-tetramethylbenzidine) substrate per well (Biorad).

### Flow cytometry and immuno-fluorescence assay

Parasite cultures at mid/late trophozoite stages were purified using VarioMACS and washed twice with PBS 0.2 % BSA. For each assay, 0.5 × 10^6^ IEs were incubated with purified rabbit anti-IT4-VAR19 and purified anti-VAR2CSA antibodies [28] diluted 1:100 in PBS 0.2 % BSA, and mouse anti-human IgM antibodies (ProMab) diluted 1:50 in PBS 0.2 % BSA. After 1-h incubation at 4 °C, the IEs were washed twice with PBS 0.2 % BSA and incubated at 4 °C for 30 min with a PE-conjugated goat F(ab’)_2_ anti-rabbit or anti-mouse IgG (Beckman Coulter, diluted 1:100 in PBS 0.2 % BSA). Cells were fixed overnight with paraformaldehyde (PFA) 4 % and washed twice with PBS. Cells were analysed by flow cytometry as described below and by confocal microscopy (Zeiss LSM700 confocal microscope).

### Flow cytometry-based binding assay

For each assay, 0.5 × 10^6^ HBEC-5i cells were incubated with 100 µg/ml of IT4-VAR19 recombinant proteins (VAR19-NTS-DBLγ6 and VAR19-CIDRα1.1) diluted in PBS 0.2 % BSA. After 30 min of incubation on ice, the cell suspension was washed twice with PBS 0.2 % BSA and incubated for 30 min with a mouse anti-His antibody (QIAGEN), at 2 μg/ml in PBS 0.2 % BSA. The cell suspension was washed twice and incubated for 30 min with PE-conjugated goat anti-mouse IgG (Beckman Coulter, diluted 1:100 in PBS 0.2 % BSA). Cells were fixed overnight with PFA 4 % and washed twice with PBS. Data acquisition was performed using a BD FACScanto II flow cytometer (Becton–Dickinson, San Jose, CA, USA) and data were analysed using the FLOWJO 8.1 software (Tree Star Inc).

### Surface plasmon resonance

The interaction between the EPCR and IT4-VAR19 recombinant proteins was studied by surface plasmon resonance (SPR) using a Biacore X100 instrument (GE Healthcare). All experiments were performed in HBS-EP buffer (GE Healthcare) at 25 °C. Recombinant EPCR_H_ was immobilized on the analysis Fc2 channel of a CM5 chip (GE Healthcare) by amine coupling to a total loading of 1324 RU. Reference channel Fc1 was blocked with 1 M ethanolamine-HCl pH 8.5 using the same chemistry. IT4-VAR19 recombinant proteins were injected for 180 s with a dissociation time of 400 s. The highest concentration was 1 µM for VAR19-NTS-DBLγ6 or 2 µM for VAR19-CIDRα1.1 and seven two-fold serial dilutions were also injected. Between the injections, the chip surface was regenerated with a 60-s pulse of 10 mM NaOH. The specific binding response to EPCR was obtained by subtracting the response given by the analytes on Fc2 by the response on Fc1. The kinetic sensorgrams were fitted to a global 1:1 interaction Langmuir model using the manufacturer’s software (Biacore X100).

### *Var* gene transcriptional profiling

*Var* gene transcriptional profiling of IEs was performed as previously described [29]. In brief, total RNA was extracted from synchronized ring stage parasites ≤10-h post-invasion, using TRIzol (Invitrogen) as recommended by the manufacturer. Total RNA was treated with TURBO DNase I (Ambion) to degrade contaminating DNA and cDNA was reverse transcribed using random hexamers and the SuperScript III First-Stand Synthesis System (Invitrogen). Five micrograms of starting total RNA were used to compare the full set of primers. Quantitative real-time PCR reactions were performed on a CFX96 thermocycler (BioRad) in 20 µl using Advanced Universal SYBR Green Supermix (BioRad) and primer pairs specific for each IT4 *var* gene [29]. The relative transcription was determined by normalization with the housekeeping control gene seryl-tRNA synthetase [PlasmoDB: PF07_0073] and converted to relative copy numbers.

### Adhesion assays

Adhesion assays on immobilized receptors were performed as described [30]. Briefly, petri dishes (Falcon 1058) with 0.5-cm diameter spots were coated overnight at 4 °C with either 10 µl of PBS containing 50 µg/ml recombinant human CD36 (R&D Systems), 50 µg/ml recombinant human ICAM-1 (R&D Systems), 1 mg/ml CSA sodium salt from bovine trachea (Sigma), 50 µg/ml recombinant EPCR or 1 % BSA. After coating, the spots were washed with PBS and blocked with 20 µl of PBS 1 % BSA for 1 h at RT. After removing the blocking solution, the spots were washed twice with PBS and 10 µl of purified IEs at trophozoite stage (25 × 10^6^ IEs/ml) were allowed to adhere. After incubating 1 h at RT, unbound IEs were washed away by adding 25 ml of PBS four times at the centre of the dish. Bound IEs were then fixed with 2 % glutaraldehyde in PBS for 2 h at RT. For each condition, the number of adherent IEs was counted microscopically using a Nikon Eclipse Ti microscope in five different fields of duplicated spots. Adhesion data presented in this study represent at least three independent experiments.

Inhibition assays of IEs on immobilized EPCR were carried out as described above with the exception of an additional pre-incubation step in which IEs were incubated for 30 min at 37 °C with serial dilutions of purified anti-IT4-VAR19 antibodies (from 0.03 to 120 µg/ml) before being allowed to adhere to EPCR-coated spots. Binding inhibition was calculated as a percentage of adhesion compared to control spots where no anti-IT4-VAR19 was added.

Adhesion assays of IEs on HBEC5i were carried out using a similar protocol as already described [12]. Briefly, HBEC-5i were cultured in 24 wells culture plates (0.4 × 10^6^ cells per well) 2 days before the assays and allowed to grow to confluency. Ten µl of IEs (1.5 × 10^6^ IEs/ml) were pre-incubated with purified anti-IT4-VAR19 antibodies (30 µg/ml) for 10 min at RT before being allowed to adhere to HBEC-5i-coated wells for 1 h at 37 °C. Unbound IEs were washed away by five washes with cell culture medium without serum. Cells were fixed with 1 % glutaraldehyde in PBS for 30 min and stained with Giemsa for 5 min. IE binding to HBEC-5i was analysed with a Nikon Eclipse Ti microscope.

### Study populations

This cohort study was conducted during 2013 and 2014 malaria transmission seasons (June to September and May to July, respectively) in Cotonou, Benin [21]. Children under 6 years of age admitted to Hôpital Mère-Enfant de la Lagune, Centre National Hospitalier Universitaire Hubert Koutoukou Maga and Hôpital Suru-Léré with malaria symptoms were screened using a rapid diagnostic test (DiaQuick Malaria *P. falciparum* Cassette, Dialab). Cerebral malaria was defined as a microscopically confirmed *P. falciparum* infection and a Blantyre coma score ≤2, with no other known cause of coma. Severe malaria was defined as a *P. falciparum* infection presenting high parasitaemia levels (>250,000 parasites/μL) or severe anaemia (haemoglobin level <5 g/dL). Uncomplicated malaria was defined, as described by WHO [31], by a *P. falciparum* infection accompanied with fever, headache or myalgia without signs of severity and/or evidence of vital organ dysfunction. Blood samples were collected when patients were admitted at the hospital and 30 days after. Children were treated according to Benin Ministry of Health guidelines.

### Ethical statement

This study was approved by the Ethical Committee of the Research Institute of Applied Biomedical Sciences (CER-ISBA), Cotonou, Benin. Children were enrolled in this study after obtaining informed and written consent from a parent or guardian, if they were diagnosed with either cerebral malarial, severe malaria or uncomplicated malaria.

### Immune recognition assay

ELISA plates (Nunc) were coated with 50 µl per well of IT4-VAR19 recombinant proteins (VAR19-NTS-DBLγ6 and VAR19-CIDRα1.1), AMA1 and VAR2CSA, at 1 µg/ml diluted in PBS, and incubated at 4 °C overnight. After coating, the wells were blocked with PBS 4 % BSA, 100 µl per well, at 37 °C for 1 h. After removing the blocking solution, 50 µl of sera was added, diluted 1:50 in PBS 2 % BSA, and incubated at 37 °C for 1 h. The wells were then washed three times with 150 µl of PBS 0.5 % Tween20. IgG binding was detected with a HRP-conjugated anti-human antibody (Jackson Immunoresearch), diluted 1:4000 in PBS 2 % BSA. The plates were read at 655 nm after addition of 100 µl per well of TMB (3,3′,5,5′-tetramethylbenzidine) substrate (Biorad).

### Statistical analyses

Optical density obtained by ELISA was converted into arbitrary units (AU) using the following formula: AU = 100 × (LnOD_test_ − LnOD_negative sample_)/(LnOD_positive sample_ − LnOD_negative sample_) [32]. Negative and positive plasma samples were chosen based on their reactivity to AMA1 already tested [33].

## Results

### Production of IT4-VAR19 and EPCR recombinant proteins

**Fig. 1:**
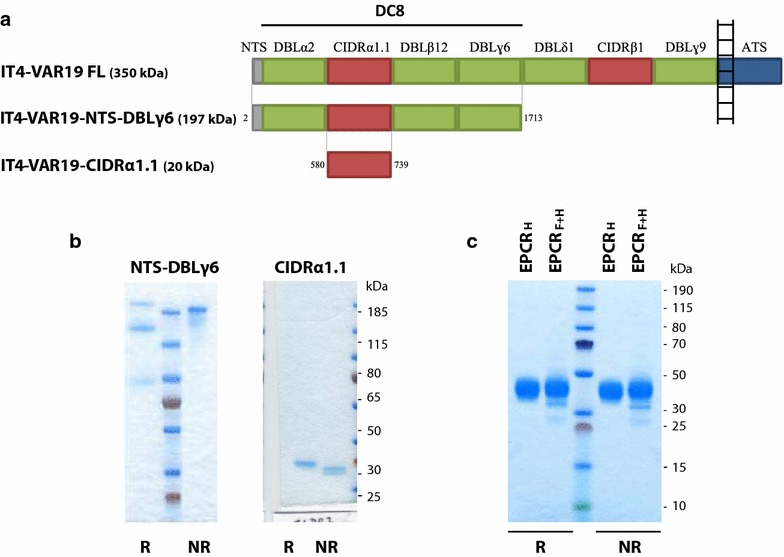
Recombinant proteins expressed in HEK293 cells. **a** Representation of IT4-VAR19 domain organization and sequence limits of the full-length IT4-VAR19 and the recombinant IT4-VAR19 domains studied (VAR19-NTS-DBLɣ6 and CIDRα1.1). IT4-VAR19 is composed of five Duffy-binding-like domains (shown in *green*), two cysteine-rich interdomain regions (shown in *red*), a transmembrane segment and an acidic C terminus sequence (ATS, shown in *blue*). **b** SDS-PAGE under reducing and non-reducing conditions of purified VAR19-NTS-DBLɣ6 and CIDRα1.1. **c** SDS-PAGE under reducing and non-reducing conditions of purified recombinant EPCR proteins (His tagged EPCR_H_ and His/FLAG tagged EPCR_F+H_)

The IT4-VAR19-DC8 cassette (VAR19-NTS-DBLγ6), composed of three DBL domains (DBLα2, DBLβ12 and DBLγ6) and a CIDR domain (CIDRα1.1), as well as the single VAR19-CIDRα1.1 domain, were produced as soluble recombinant proteins (Fig. 1a, b).

His-tagged recombinant VAR19-NTS-DBLγ6 (197 kDa) was expressed in human embryonic kidney cells (HEK 293F). After a two-step purification process, immobilized metal ion affinity chromatography (IMAC) and gel filtration, an estimated yield of 1 mg of purified recombinant protein was recovered from a litre of HEK culture (Fig. 1b). SDS-PAGE analysis of VAR19-NTS-DBLγ6 under non-reducing and reducing conditions revealed that a significant proportion of the recombinant protein was cleaved during the production phase. Indeed, even if Coomassie staining manifestly showed the presence of a single band at the expected apparent molecular weight under non-reducing conditions, two lower size extra bands (≈120 and ≈75 kDa) appeared under reducing conditions (Fig. 1b). These byproducts, which respective apparent molecular weights added up to one of the full length VAR19-NTS-DBLγ6, most likely resulted from a one-site cleavage, the protein fragments being held together by the presence of many predicted disulfide bonds in non-reducing conditions.

The recombinant his-tagged VAR19-CIDRα1.1 domain with an expected molecular weight of 20 kDa was expressed in *E. coli* SHuffle^®^ cells and purified following the same two-step process described above. The final production yield of VAR19-CIDRα1.1 was estimated at 3.8 mg of purified recombinant protein per litre of bacteria culture. SDS-PAGE with subsequent gel staining with Coomassie revealed one single band at the expected molecular weight in reducing conditions whereas two close bands were visible in non-reducing conditions (Fig. 1b).

Recombinant proteins comprising the extracellular region of the human EPCR (24 kDa), with a 6× His tag at the C-terminus (EPCR_H_) or with an additional FLAG tag at the C-terminus (EPCR_F-H_) were also produced. Proteins were expressed in HEK 293F cells as soluble proteins with a production yield of 8.4 and 2.6 mg per litre of starting culture, respectively. SDS-PAGE analysis of EPCR under non-reducing and reducing conditions indicated that no proteolytic degradation occurred during production and purification processes (Fig. 1c). Under reducing conditions, EPCR_H_ and EPCR_F-H_ migrated slightly above their expected molecular weight (≈40 kDa). The recombinant proteins were therefore considered as suitable to carry on the study.

### Kinetic binding analysis of EPCR-IT4-VAR19 interaction

**Fig. 2:**
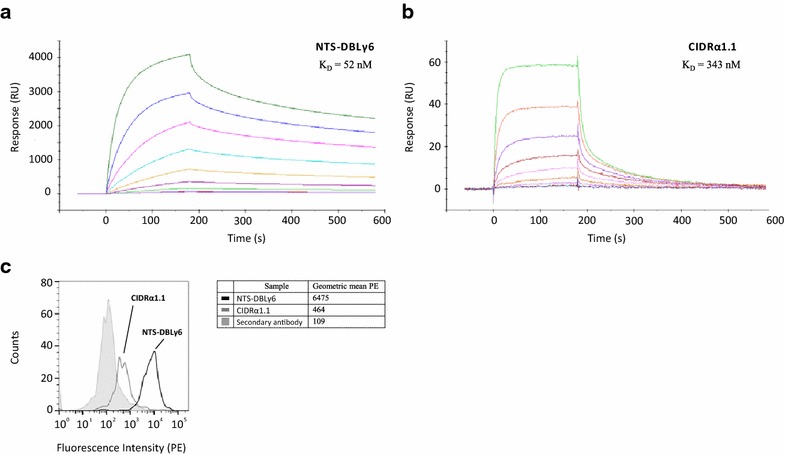
Recombinant IT4-VAR19 proteins bind specifically to EPCR and human brain endothelial cells. **a**, **b** Sensorgram showing interaction between IT4-VAR19 proteins and EPCR. Recombinant EPCR protein was immobilized on a chip and recombinant IT4-VAR19 proteins were injected at eight different concentrations from 1 µM to 7.8 nM for VAR19-NTS-DBLɣ6 (**a**) and 2 µM to 15.6 nM for CIDRα1.1 (**b**). Using the manufacturer’s software, a Langmuir model for a 1:1 interaction was chosen to evaluate the data and determine the K_D_ constants. **c** Binding assay of the IT4-VAR19 proteins (NTS-DBLɣ6 and CIDRα1.1) to HBEC5i by flow cytometry. HBEC5i cells were incubated with recombinant IT4-VAR19 proteins and binding was detected using a mouse anti-His IgG and a PE-conjugated anti-mouse IgG

The IT4-VAR19 recombinant proteins were subjected to SPR analysis to determine their affinity constants (K_D_) to recombinant EPCR_H_. The fitted kinetic data derived from the SPR sensorgrams revealed that recombinant VAR19-NTS-DBLγ6 protein had a high affinity for recombinant EPCR_H_ with a K_D_ in the nanomolar range (K_D_ = 52 nM) whereas the affinity of VAR19-CIDRα1.1 for EPCR_H_ was much lower with a K_D_ of 343 nM (Fig. 2a, b and Table 1).

**Table 1:** Affinity constants reflecting the binding of IT4VAR19 recombinant proteins to EPCR

	k_on_ (M^−1^s^−1^)	k_off_ (s^−1^)	K_D_ (M)
NTS-DBLɣ6	2.2E+04	1.1E−03	5.2E−08
CIDRα1.1	3.5 E+04	1.2E−02	3.4E−07

### Recombinant IT4-VAR19 proteins specifically bind to human brain endothelial cells

The binding of the recombinant IT4-VAR19 proteins to human brain endothelial cells (HBEC5i cell line) was assessed. Endothelial cells were lifted from tissue culture dishes and incubated with IT4-VAR19 recombinant proteins. Flow cytometry analysis revealed that both VAR19-NTS-DBLγ6 and VAR19-CIDRα1.1 bound to HBEC5i as reflected by a shift in fluorescence intensity compared to cells incubated with PBS alone (Fig. 2c). The VAR19-NTS-DBLγ6 and VAR19-CIDRα1.1 binding to HBEC5i was evaluated using the same concentrations of soluble proteins (100 μg/ml). Considering the respective sizes (197 versus 20 kDa), VAR19-CIDRα1.1 was used in a ten-times excess molarity compared to VAR19-NTS-DBLγ6. Even though these experimental conditions were favourable for VAR19-CIDRα1.1 to occupy more EPCR-binding sites at the surface of HBEC5i than for VAR19-NTS-DBLγ6, the geometrical mean fluorescence intensity resulting from VAR19-NTS-DBLγ6 interactions with the endothelial cells was 14 times greater than that of VAR19-CIDRα1.1. Taken together, these results show that IT4-VAR19 binds to HBEC5i and that this binding involves, at least to some extent, VAR19-CIDRα1.1 binding to EPCR.

### Antibodies raised against VAR19-NTS-DBLγ6 are able to inhibit EPCR binding

Two rabbits were immunized with the VAR19-NTS-DBLγ6 recombinant protein following a two-month schedule for a total of four injections. Antibody titres (IgG) at day 49 and day 63 were estimated by ELISA using the immunizing protein as target antigen (Additional file 1). VAR19-NTS-DBLγ6 appeared to be a highly immunogenic antigen as reflected by the very elevated antibody titres obtained for the two rabbits as early as day 49 (787,000 and 809,000). The pre-immune sera of both animals did not react with the recombinant protein used for immunization.

To evaluate the ability of these antibodies to inhibit recombinant IT4-VAR19 proteins adhesion to EPCR_H_, purified rabbit IgG were tested in protein binding inhibition assays. Purified IgG from both rabbits were able to inhibit the adhesion of recombinant VAR19-NTS-DBLγ6 protein to EPCR_H_ in a dose-dependent manner (Additional file 2). The inhibiting properties of IgG issued from the two different rabbits were similar even if a 10 % increased inhibition was obtained with IgG from rabbit 2 at the highest concentration tested. Purified IgG from pre-immune sera had no inhibitory activity. IgG purified from the immune serum of rabbit 2 will be used throughout the rest of study and will be referred as anti-VAR19 IgG.

### Selection of IEs on anti-VAR19 antibodies, EPCR and HBEC5i cells

Erythrocytes infected with the FCR3 (IT4) parasite strain, formerly selected for CSA-binding (FCR3-CSA) and therefore expressing VAR2CSA, were selected by successive panning on anti-VAR19 IgG, recombinant EPCR or HBEC5i cells. Panning was pursued until the same *var* gene transcription profile was observed in two consecutive panning rounds.

**Fig. 3:**
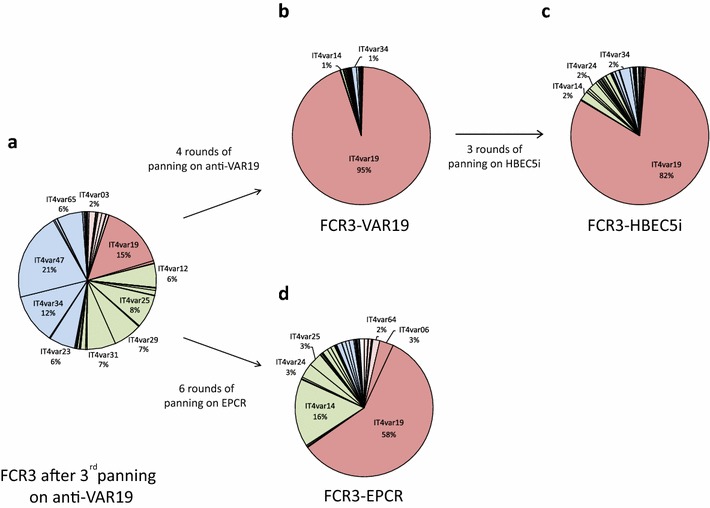
Transcriptional analysis of *var* genes expressed after panning IEs anti-IT4-VAR19 antibodies (**a**, **b**), HBEC5i (**c**) and with EPCR (**d**). *Var* genes are organized by group: group A in *pink*, group B/A in *red*, group B in *green*, group C in *blue*. Results were normalized with the control housekeeping gene seryl-tRNA synthetase (PF07_0073). The percentage of each *var* transcript is shown as pie charts

FCR3-CSA, panned for three rounds on anti-IT4-VAR19 IgG (Fig. 3a) express a highly diverse mixture of *var* transcripts from different groups including the *IT4var19* (15 %).

After seven rounds of panning on anti-VAR19 IgG, a population, referred to as FCR3-VAR19, was selected. In FCR3-VAR19, 95 % of IEs expressed the *IT4var19* transcript, 1 % the *ITvar34* and 1 % the *ITvar14* transcript, the remaining 3 % of the population expressing a panel of other *var* transcripts. The detailed *var* gene expression profiles of infected erythrocytes panned three times and seven times are depicted in Fig. 3a, b and Additional file 3A and B, reflecting the progressive enrichment of IEs expressed the *IT4var19* transcript.

The selected FCR3-VAR19 IEs were then reselected on human brain endothelial cells (HBEC5i). After two and three panning rounds, the same *var* gene transcription profile was observed in which 82 % of IEs expressed the *IT4var19* transcript (Fig. 3c and Additional file 3D). This selected population of IEs is referred to as FCR3-HBEC5i. FCR3-HBEC5i IEs also expressed a mixture of group B *var* transcripts (2 % *IT4var14* (DC17), 2 % *IT4var24* as well as 2 % of the group C *IT4var34* transcript).

In a second set of experiments, FCR3-CSA, panned for three rounds on anti-VAR19 IgG (Fig. 3a) was subsequently selected on recombinant EPCR_H_. Following six extra rounds of panning on EPCR_H_, a population containing 58 % of IEs expressing *IT4var19* transcript was selected (Fig. 3d and Additional file 3C). This selected population of IEs, referred to as FCR3-EPCR IEs, also expressed a mixture of group B *var* transcripts (16 % *IT4var14* (DC17), 3 % *IT4var24*, 3 % *IT4var25*) as well as 3 % of the group B/A *IT4var6* transcript, a *var* gene that encodes for a PfEMP1 possessing, as IT4-VAR19, a DC8 domain cassette.

### Adhesion phenotype of selected IEs

**Fig. 4:**
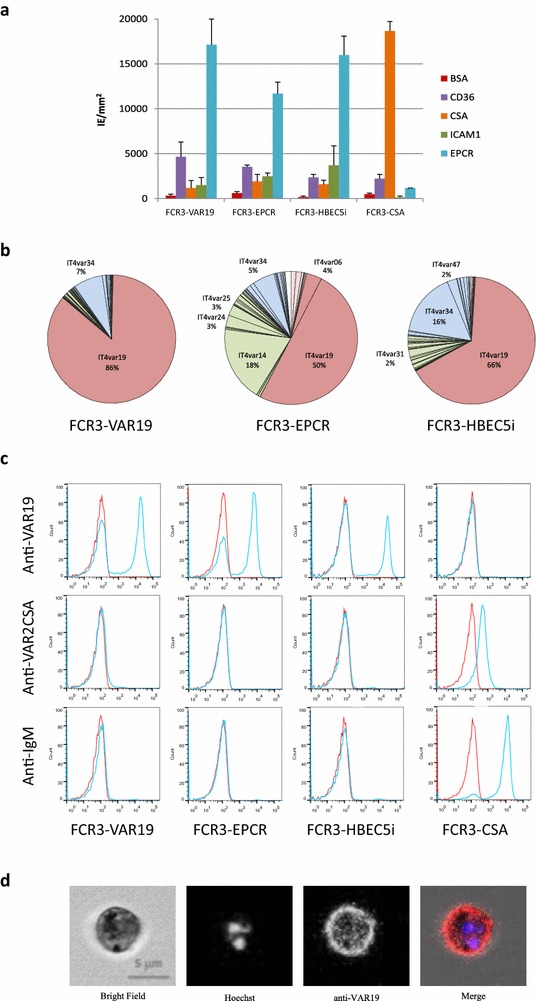
**a** Adhesion profile of IEs selected with anti-VAR19 antibodies (FCR3-VAR19), EPCR (FCR3-EPCR) and human brain endothelial cells (FCR3-HBEC5i). Data shown is the mean number of IEs per mm^2^ adhering to receptors coated on plastic Petri dishes, as determined in three independent assays in duplicated spots (*error bars* represents the standard deviation). **b** Transcription analysis of *var* genes expressed at the same parasite cycle as the adhesion assays were done. **c** Flow cytometry analysis of IEs obtained after panning labelled with rabbit anti-VAR19, anti-VAR2CSA and mouse anti-IgM antibodies (*blue* histograms) compared to negative control (without primary antibody, *red* histograms). **d** Immunofluorescence assay of FCR3-VAR19 IEs stained with rabbit anti-VAR19 IgG (*red*). The parasite nucleus is stained with Hoechst 3342 (*blue*)

Static binding assays, using recombinant proteins or sugars coated on plastic (CD36, ICAM-1, EPCR_H_ or CSA), were performed to determine the binding phenotype of FCR3-VAR19, FCR3-EPCR and FCR3-HBEC5i IEs. The former erythrocyte population FCR3-CSA, known to adhere to CSA and not to any other receptors, was also included in the assay as a reference. The three selected IE populations revealed a strong binding to EPCR_H_ (Fig. 4a) and much lower adhesion to other host cytoadhesion receptors (CD36, ICAM1 and CSA). Importantly, by comparison to other selected IE populations, ICAM1 binding was higher in the FCR3-HBEC5i IE population. For each IE population, the *var* transcript profile was verified the day before the adhesion phenotypes were assessed (Fig. 4b).

Flow cytometry experiments showed that FCR3-VAR19, FCR3-EPCR and FCR3-HBEC5i IEs were highly recognized by anti-VAR19 IgG whereas the control IE population FCR3-CSA was not (Fig. 4c). Anti-VAR2CSA antibodies, recognizing FCR3-CSA, did not react with any of the newly selected IE populations. Unlike for FCR3-CSA, neither FCR3-VAR19, FCR3-EPCR nor FCR3-HBEC5i IEs were able to fix non-immune IgM.

In a qualitative analysis, anti-IT4-VAR19 IgG staining of FCR3-VAR19 IEs revealed a dotty fluorescence pattern associated to the red cell membrane (Fig. 4d), similar to those obtained when targeting other PfEMP1 proteins expressed at the IE surface [34].

### VAR19-NTS-DBLɣ6 antibodies inhibits the adhesion of FCR3-VAR19 and FCR3-EPCR IEs to EPCR

**Fig. 5:**
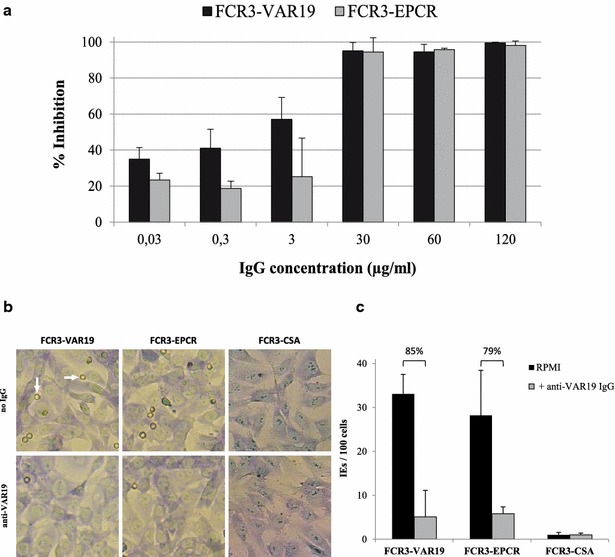
Static inhibition assay of IEs on immobilized EPCR and human brain endothelial cells. **a** Adhesion inhibition assays on EPCR-coated plastic spots were performed using FCR3-VAR19 and FCR3-EPCR IEs pre-incubated with anti-VAR19 antibodies at different concentrations (ranging from 0.03 to120 µg/ml). The percentage of inhibition was calculated using the number of IEs obtained after pre-incubation with PBS alone as a reference. Error bars represent the standard deviation of three independent experiments. **b** Adhesion of FCR3-VAR19, FCR3-EPCR and FCR3-CSA IEs to HBEC5i cells in the presence or absence of anti-VAR19 antibodies is shown. *Arrows* points to adherent IEs. **c** Mean number of IEs adhering per 100 HBEC5i cells was determined in two independent assays (*error bars* represents the standard deviation). The percentage of inhibition was calculated using the number of IEs obtained after pre-incubation with PBS alone (no IgG) as a reference

The capacity of anti-VAR19 antibodies to inhibit the adhesion of FCR3-VAR19 and FCR3-EPCR IEs to EPCR_H_ was evaluated. A static binding inhibition assay where recombinant EPCR_H_ was immobilized on plastic, revealed that anti-VAR19 antibodies were able to inhibit the adhesion of FCR3-VAR19 and FCR3-EPCR IEs to EPCR_H_, in a dose-dependent manner (Fig. 5a). FCR3-VAR19 binding to EPCR was inhibited to a higher extent than FCR3-EPCR binding at low antibody concentrations.

### VAR19-NTS-DBLɣ6 antibodies partially inhibit the adhesion of FCR3-VAR19 and FCR3-EPCR IEs to HBECs

Both FCR3-VAR19 and FCR3-EPCR IEs were able to adhere to HBECs in static binding assays (Fig. 5b). No binding of FCR3-CSA IEs was observed on HBEC5i. In order to evaluate the capability of antibodies raised against VAR19-NTS**-**DBLɣ6 to interfere with FCR3-VAR19 and FCR3-EPCR IE binding to HBEC5i cells, both selected IE populations were pre-incubated with 30 µg/ml of antibody before being allowed to interact with a monolayer of endothelial cells. Anti-VAR19 IgG were able to partially inhibit the binding of FCR3-VAR19 (85 % inhibition) and FCR3-EPCR (79 % inhibition) IEs (Fig. 5c).

### IT4-VAR19-DC8 cassette and CIDRα1.1 are not associated with increased recognition of convalescent-phase sera from Beninese children

**Fig. 6:**
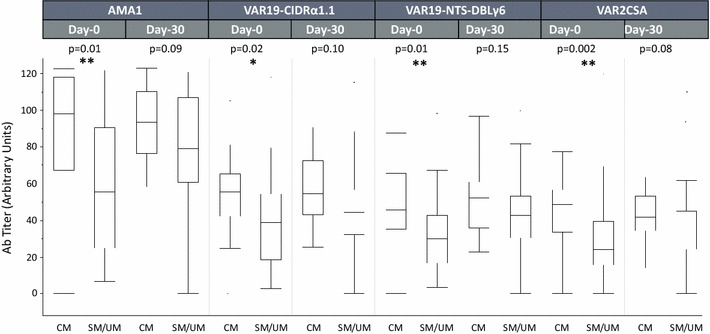
Antibody levels to IT4-VAR19 proteins (NTS-DBLɣ6 and CIDRα1.1), VAR2CSA and AMA1 in the plasma of children from Benin presenting with cerebral malaria (CM, N = 18) or with severe or uncomplicated malaria (SM/UM, N = 34) attack. Plasma samples were collected at admission (day 0) and 30 days later (day 30)

The evaluation of the antigenic relationship of DC8-PfEMP1 variants, was assessed by quantifying the levels of naturally acquired antibody targeting the IT4-VAR19 full-length DC8 cassette, the single EPCR-binding CIDRα1.1 domain, the apical membrane antigen (AMA)-1 and full length VAR2CSA extracellular region, in plasma samples from Beninese children presenting either acute mild malaria (UM), severe malaria (SM) or cerebral malaria (CM) at the time of their admission to the clinic and from convalescent-phase sera collected 30 days after anti-malaria treatment (Fig. 6). In a first analysis, no significant difference in immune recognition was observed between the SM and UM groups, for all the recombinant proteins tested. It was therefore decided to combine and compare those two groups (SM/UM) with the CM group alone. Interestingly, children with CM had significantly higher levels of IgG towards all the different antigens examined compared to the SM/UM group at day 0 (p values <0.05) but not at day 30. Surprisingly, no significant difference in the levels of antibodies towards IT4-VAR19 antigens was observed in both groups between plasma samples collected during the acute malaria phase compared to samples collected 30 days after anti-malaria treatment.

## Discussion

The human EPCR, which is the cellular receptor for protein C, is expressed by a variety of endothelial cells as a membrane-associated protein but also exists as a soluble form, displaying multiple and important physiological functions [18]. The 1 TM (trans-membrane domain) EPCR is a glycosylated protein that possesses two cysteines (C118–C186) engaged in a disulfide bond [35]. This article describes a method of expression of the EPCR topological domain (aa 18–210) in a human-based system supporting type I N-glycolsylation and disulfide bond formation as well as an efficient two-step purification procedure. The qualitative analysis of the two differently tagged versions of EPCR revealed that the recombinant proteins most likely exhibit the structural and functional features similar to that of native EPCR. Recombinant EPCR_H_ and EPCR_F-H_ migrated on SDS-PAGE to an apparent molecular weight of ≈40 kDa, higher than their theoretical weights of 23.7 and 24.7 kDa, respectively (Fig. 2). This is consistent with the fact that post-translational modifications, such as the four expected *N*-glycosylations (positions 47, 64, 136, 172), occurred during the expression process.

Turner et al. provided the first experimental evidence that a DC8 cassette-carrying PfEMP1 (IT4-VAR20), produced as a full-length recombinant protein, was able to bind to EPCR with high affinity [15], this binding being only mediated by the CIDRα1.1 single domain. Despite extensive sequence diversity, CIDR domains belonging to the DC8 and DC13 cassettes appear to retain their ability to bind to EPCR [16, 36] with some reported exceptions, such as the DC8-CIDRα1.6 [PlasmoDB: PF08_0140] of the 3D7 parasite strain [15].

This work shows that the multi-domain VAR19-NTS-DBLγ6 binds to EPCR with a greater affinity than the CIDRα1.1 domain alone (Figs. 2, 3). Indeed, the K_D_ of 52 nM reflecting the affinity of VAR19-NTS-DBL3γ for EPCR is almost seven times lower than that of the CIDRα1.1 domain alone (K_D_ = 343 nM). The only other study comparing the affinity for EPCR binding of a DC8-type multi-domain construct (full length IT4-VAR20) and the corresponding CIDR domain alone with the same experimental settings, reported dissociation constants of 10 nM and 29 nM, respectively [15]. The lower observed affinity could be due to the fact that the CIDRα1.1 construct used in this study was slightly shorter than previously published [12, 36].

This work also demonstrates that VAR19-NTS-DBLγ6 binding to the EPCR-expressing endothelial cell line (HBEC5i) is more pronounced than that of the CIDRα1.1 domain alone (Fig. 2). This is consistent with our previous observation with EPCR (Fig. 3). In addition, recent data showed that all seven individual domains of IT4-VAR19, including CIDRα1.1, were able to bind to diverse endothelial cells [16]. Therefore, the binding of VAR19-NTS-DBLγ6 to HBEC5i may result from (i) interactions of CIDRα1.1 with EPCR, and from (ii) synergic interactions from other domains (DBLα2, DBLβ12, DBLγ6) with (one) other, yet unknown, cellular receptor(s). In this context, the *var* gene expression profiles of IEs after selection on either anti-VAR19 antibodies (raised against VAR19-NTS-DBLγ6), recombinant EPCR or HBEC5i cells was assessed. After panning with anti-VAR19 IgG, an homogenous IE population (95 %) expressing the *IT4var19* transcript (FCR3-VAR19) was obtained, whereas selection on EPCR led to a more heterogeneous population expressing IT4var19 (58 %) but also a variety of other *var* genes belonging to the group B *IT4var14*, *IT4var24*, *IT4var25* displaying other domain cassette types than DC8 or DC13 (Fig. 3). IT4-VAR19 represent the preferentially expressed PfEMP1 when FCR3 IEs are selected based on their capability to bind EPCR. No significant population of IEs expressing the EPCR-binding IT4-VAR20 PfEMP1 (<1 %) was found. Furthermore, only 3 % of FCR3-EPCR IEs were expressing the EPCR-binding IT4-VAR06 PfEMP1. However, a significant proportion of IEs (16 %) expressing IT4-VAR14-DC17 were selected upon panning to EPCR. Interestingly, IT4-VAR14 possesses a CD36-binding CIDRα5 and an ICAM-1-binding DBLβ type [37] and was previously reported to weakly bind EPCR when binding was assessed using the CIDR1α5 domain alone [15]. Furthermore, IT4-VAR14-DC17 IEs had weak binding to the immortalized human brain microvascular endothelial cell line (THBMEC) relative to IT4-VAR19 [12]. Taken together, these results may suggest that IT4-VAR14-DC17 IEs can bind to EPCR and endothelial cells even though this PfEMP1 variant possesses a CD36-binding CIDRα5. Interestingly, no major change of transcription profile was found after three rounds of panning of the FCR3-VAR19 population on HBEC5i endothelial cells. By comparison to the study by Claessens et al. [13], the *IT4var07* gene was not upregulated after three pannings on HBEC5i cells. The three selected IE populations revealed a strong binding to EPCR (Fig. 4a) and a much lower adhesion to other host receptors (CD36, ICAM1 and CSA). Taken together, these results indicate that IT4-VAR19 is the preferentially selected EPCR binding PfEMP1 from the IT4 strain.

Antibodies raised against VAR19-NTS-DBLɣ6 DC8 cassette were able to fully inhibit the adhesion of FCR3-VAR19 and FCR3-EPCR IEs to EPCR, in a dose-dependent manner (Fig. 5a) but partially inhibit the adhesion of FCR3-VAR19 and FCR3-EPCR IEs to HBEC5i (Fig. 5c). These results suggest that the adhesion of these parasite lines is largely dependent on EPCR interaction but that other interactions involving other PfEMP1 domains than CIDRα1.1, with as yet unidentified host receptors, are involved in the adhesion to endothelial cells. Furthermore, as only 50 % of the FCR3-EPCR IEs population express *IT4var19* transcript (Fig. 4b), it is likely that the sera raised against VAR19-NTS-DBLɣ6 DC8 cassette cross-reacted with the other EPCR binding variants.

The evaluation of the role of IT4-VAR19-like EPCR binding parasites in severe childhood infections was assesses by quantifying the levels of naturally acquired antibody to the IT4-VAR19 recombinant proteins in plasma samples from Beninese children, presenting either UM, SM or CM. No difference in the plasma level of antibodies was observed between VAR2CSA and VAR19 antigens. Only antibodies to VAR19-NTS**-**DBLɣ6 slightly, although not significantly, increased between admission and convalescence. These results suggest that children did not develop humoral immunity against the IT4-VAR19 DC8 cassette and therefore that PfEMP1s expressed by IEs infecting children in Benin are antigenically distinct from IT4-VAR19 DC8 cassette. Although recent evidence indicates that parasites expressing DC8 and DC13 domain cassettes are increased in severe paediatric malaria infections [14, 38], further studies are needed to validate the exact role of both domain cassettes in severe malaria conditions, and how immunity develops against those variant antigens. Surprisingly the pregnancy associated malaria antigen VAR2CSA was also recognized by children sera although this *var* gene is supposed to be only expressed during placental malaria. It was previously reported in different studies that specific IgG against VAR2CSA are present at significant levels among some men and children [39, 40], suggesting that exposure to these variants is not exclusive to pregnancy. Although the level and prevalence of reactivity to VAR2CSA is significantly higher in pregnant multigravid women, the reactivity in plasma from children emphasizes our incomplete understanding of the protective immune response during placental malaria and childhood. Further investigations are needed to assess the exact role of VAR2CSA in severe malaria pathogenesis and immunity. Finally, the only significant difference observed was that children with CM had significantly higher levels of IgG to all antigens examined compared to SM/UM children. This result confirms previous observations that children with CM are distinguished by higher antibody levels to all antigens tested [41].

## Conclusions

These data indicate that IT4-VAR19 is the preferentially selected IT4-EPCR-binding PfEMP1 but that humoral immunity against the EPCR-binding VAR19-DC8 cassette or the CIDRα1.1 domain is not boosted during a single episode of severe malaria in Benin.

## Additional files

10.1186/s12936-015-1008-5 Rabbit antibody titers (IgG) following animal immunization with VAR19-NTS-DBLγ6.
10.1186/s12936-015-1008-5. Antibodies raised against VAR19-NTS-DBLγ6 inhibit its interaction with EPCR.
10.1186/s12936-015-1008-5 Transcriptional analysis of *var* genes expressed after panning IEs anti-IT4-VAR19 antibodies (A and B), HBEC5i (C) and with EPCR (D). *Var* genes are organized by group: group A in pink, group B/A in red, group B in green, group C in blue. Results were normalized with the control housekeeping gene seryl-tRNA synthetase (PF07_0073).
