# Erratum to: Beninese children with cerebral malaria do not develop humoral immunity against the IT4-VAR19-DC8 PfEMP1 variant linked to EPCR and brain endothelial binding

**DOI:** 10.1186/s12936-015-1061-0

**Published:** 2016-01-11

**Authors:** Sofia Nunes-Silva, Sébastien Dechavanne, Azizath Moussiliou, Natalia Pstrąg, Jean-Philippe Semblat, Stéphane Gangnard, Nicaise Tuikue-Ndam, Philippe Deloron, Arnaud Chêne, Benoît Gamain

**Affiliations:** Inserm UMR_1134, Paris, France; Université Paris Diderot, Sorbonne Paris Cité, UMR_S1134, Paris, France; Institut National de la Transfusion Sanguine, 6 rue Alexandre Cabanel, Paris, 75015 France; Laboratory of Excellence GR-Ex, Paris, France; Institut de Recherche pour le développement, UMR_216, Mère et enfant face aux infections tropicales, Paris, France; Faculté de pharmacie, PRES Sorbonne Paris Cité, Paris, France

## Erratum to: Malar J (2015) 14:493 DOI 10.1186/s12936-015-1008-5

After publication of the original article [1], it was noticed that Fig. 1 was inadvertently altered during production. While ensuring the Figure was suitable for inclusion and conformed to style guidelines, Fig. 1b was mistakenly cropped in a way that reduced the effectiveness of the Figure. Figure 1 has now been replaced with the correct version in the original article, and is published correctly below.

**Fig. 1:**
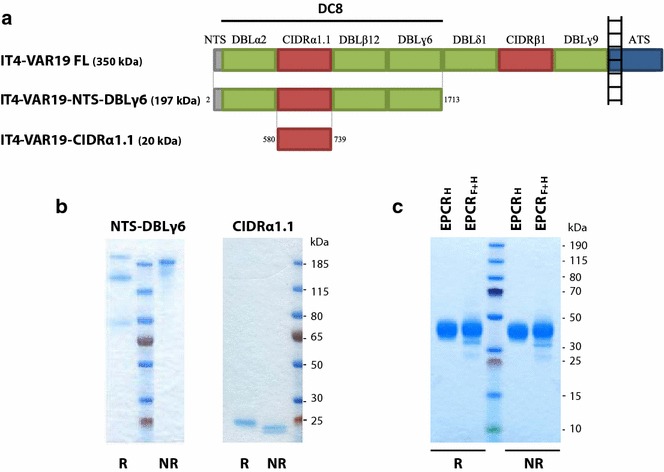
Recombinant proteins expressed in HEK293 cells. **a** Representation of IT4-VAR19 domain organization and sequence limits of the full-length IT4-VAR19 and the recombinant IT4-VAR19 domains studied (VAR19-NTS-DBLɣ6 and CIDRα1.1). IT4-VAR19 is composed of five Duffy-binding-like domains (shown in *green*), two cysteine-rich interdomain regions (shown in *red*), a transmembrane segment and an acidic C terminus sequence (ATS, shown in *blue*). **b** SDS-PAGE under reducing and non-reducing conditions of purified VAR19-NTS-DBLɣ6 and CIDRα1.1. **c** SDS-PAGE under reducing and non-reducing conditions of purified recombinant EPCR proteins (His tagged EPCR_H_ and His/FLAG tagged)

Additionally, Additional file 2 was incorrectly supplied as a duplicate of Additional file 1 during the proofing process. The original article has been updated with the correct file for Additional file 2, ‘Antibodies raised against VAR19-NTS-DBLγ6 inhibit its interaction with EPCR’, which also appears correctly in this erratum.

## Additional file

10.1186/s12936-015-1061-0 Antibodies raised against VAR19-NTS-DBLγ6 inhibit its interaction with EPCR.
