# Supplementary material for: Beninese children with cerebral malaria do not develop humoral immunity against the IT4-VAR19-DC8 PfEMP1 variant linked to EPCR and brain endothelial binding
Source: Malar J. 2015 Dec 8;14:493. doi: 10.1186/s12936-015-1008-5 (PMC4672576; doi:10.1186/s12936-015-1008-5)
Supplement: Supplementary file 2 — 10.1186/s12936-015-1008-5. Antibodies raised against VAR19-NTS-DBLγ6 inhibit its interaction with EPCR. [file 12936_2015_1008_MOESM2_ESM.pdf]

Supplementary Table 1

Antibodies raised against VAR19-NTS-DBLγ6 inhibit its interaction with EPCR

Rabbit	Day 0	Day 49	Day 63
1	< 50	787,000	558,000
2	< 50	809,000	756,000
