# Supplementary figures and images for: Beninese children with cerebral malaria do not develop humoral immunity against the IT4-VAR19-DC8 PfEMP1 variant linked to EPCR and brain endothelial binding
Source: Malar J. 2015 Dec 8;14:493. doi: 10.1186/s12936-015-1008-5 (PMC4672576; doi:10.1186/s12936-015-1008-5)

Supplementary Figure 2

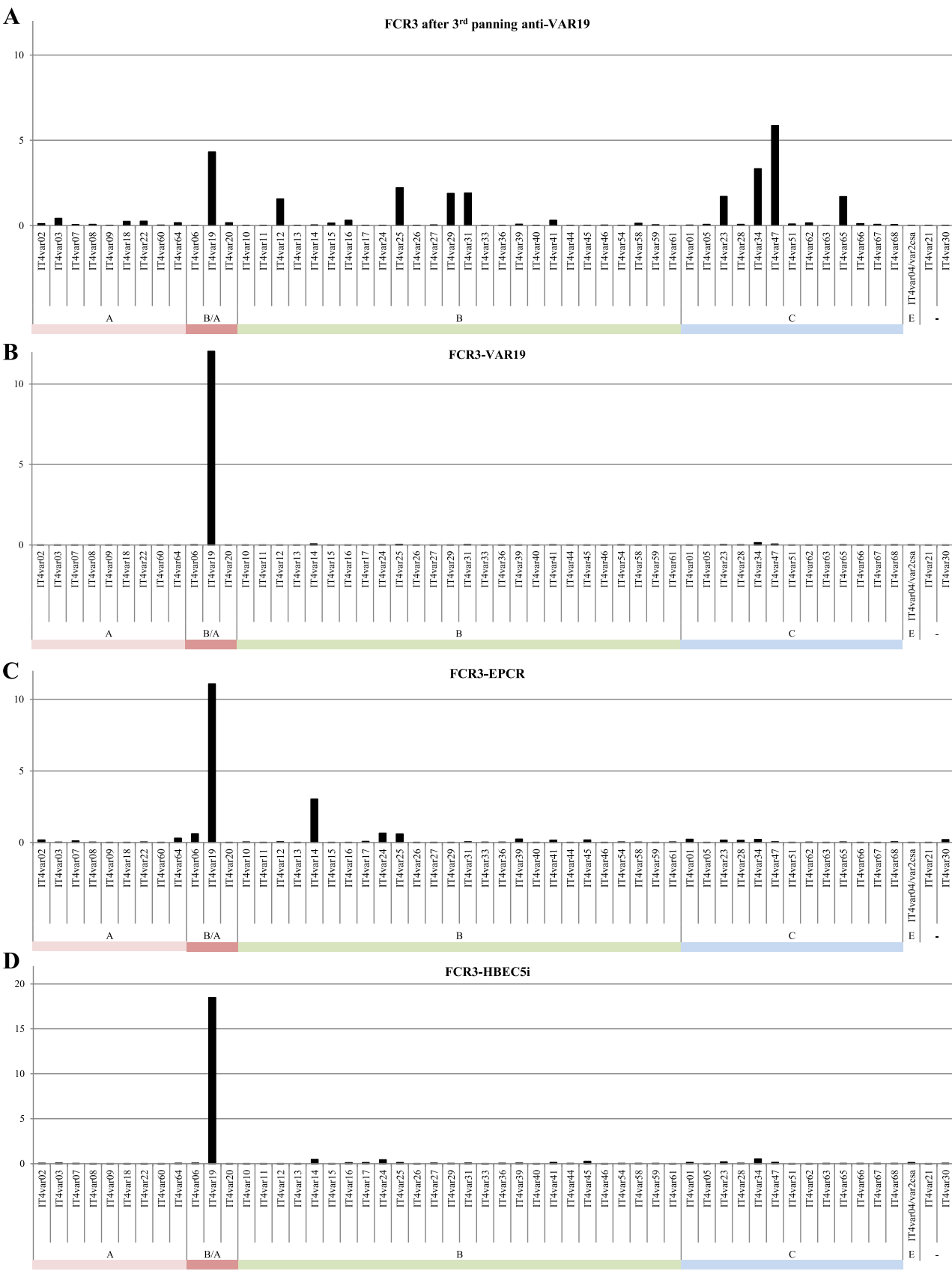

Supplement: Supplementary file 3 — 10.1186/s12936-015-1008-5 Transcriptional analysis of var genes expressed after panning IEs anti-IT4-VAR19 antibodies (A and B), HBEC5i (C) and with EPCR (D). Var genes are organized by group: group A in pink, group B/A in red, group B in green, group C in blue. Results were normalized with the control housekeeping gene seryl-tRNA synthetase (PF07_0073). [file 12936_2015_1008_MOESM3_ESM.pdf]
