# Supplementary figures and images for: Erratum to: Beninese children with cerebral malaria do not develop humoral immunity against the IT4-VAR19-DC8 PfEMP1 variant linked to EPCR and brain endothelial binding
Source: Malar J. 2016 Jan 11;15:22. doi: 10.1186/s12936-015-1061-0 (PMC4710028; doi:10.1186/s12936-015-1061-0)

# Supplementary Figure 1

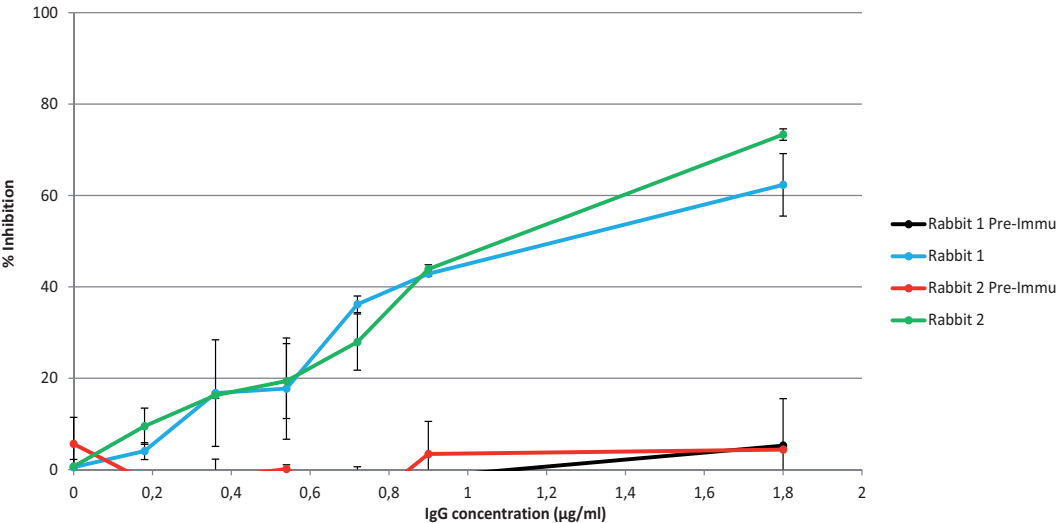

Supplement: Supplementary file 1 — 10.1186/s12936-015-1061-0 Antibodies raised against VAR19-NTS-DBLγ6 inhibit its interaction with EPCR. [file 12936_2015_1061_MOESM2_ESM.pdf]
